# A Phase II Study of Neoadjuvant Chemoradiotherapy with Docetaxel, Cisplatin and 5-FU Followed by Surgical Resection in the Treatment of Locally Advanced Esophagogastric Junction Cancer and Locally Advanced Esophageal Cancer

**DOI:** 10.3390/clinpract14020051

**Published:** 2024-04-22

**Authors:** Chien-Chih Chen, Hui-Ling Yeh, Cheng-Yeh Chuang, Chung-Ping Hsu

**Affiliations:** 1 Department of Radiation Oncology, Taichung Veterans General Hospital, Taichung 40705, Taiwan; dingjin@vghtc.gov.tw; 2 Department of Medical Imaging and Radiological Sciences, Central Taiwan University of Science and Technology, Taichung 40601, Taiwan; 3 Division of Thoracic Surgery, Department of Surgery, Taichung Veterans General Hospital, Taichung 40705, Taiwan; chuang5045@vghtc.gov.tw; 4 Department of Thoracic Surgery, Hualien Tzu Chi Medical Hospital, No. 707 Sec. 3, Zhongyang Rd., Hualien City 970473, Taiwan

**Keywords:** chemoradiotherapy, gastrointestinal cancer, neoadjuvant therapy

## Abstract

Purpose: We conducted a phase II study evaluating chemoradiotherapy in patients with advanced esophageal cancer, using the docetaxel, cisplatin, and 5-fluorouracil (DCF) regimen followed by surgery. The primary purposes of this clinical trial were to assess the efficacy and safety of chemoradiotherapy employing the DCF regimen in the treatment of advanced esophageal cancer. Material and methods: We enrolled a total of 24 newly diagnosed esophageal cancer patients between April 2015 and November 2017 in this prospective study. The radiotherapy regimen consisted of a total dose of 45 Gy in 25 fractions. The chemotherapy protocol included docetaxel 35 mg/m^2^ for 1 h on day 1 and day 29, cisplatin 35 mg/m^2^ for 1 h on day 1 and day 29, and 5-FU 400 mg/m^2^ for 24 h on day 1–4 and day 29–32. The patients who accepted the re-staging exam should undergo surgery in 4–8 weeks after the completion of radiotherapy. The primary endpoints of this study were disease-free survival (DFS), overall survival (OS), and the evaluation of hematologic toxicity. Results: The study population had a median age of 55.5 years, ranging from 44 to 66, with over 90% of the patients being male. The 5-year DFS was 37.1%, and the 5-year OS was 48.7%. The pathologic complete response rate was 45.8% (11/24). The most common types of toxicity were leukopenia and thrombocytopenia. No grade 3 or greater hematologic toxicity was reported. Conclusions: The use of the DCF regimen in neoadjuvant chemoradiotherapy followed by surgery demonstrated tolerable toxicity and achieved acceptable DFS and OS outcomes.

## 1. Introduction

Esophageal cancer is a common malignancy globally, characterized by a notable mortality rate [1]. Neoadjuvant chemoradiotherapy has been established as a cornerstone therapeutic approach for locally advanced esophageal cancer, aimed at enhancing the clinical outcomes of surgical resection [2,3,4]. The primary goals of neoadjuvant chemoradiotherapy encompass tumor regression, facilitating complete resection, and potentially augmenting survival rates in patients with locally advanced esophageal cancer. This therapeutic strategy is formulated to target micrometastatic disease, enhance local control, and potentially eradicate residual microscopic lesions. The integration of both chemotherapy and radiotherapy in neoadjuvant treatment is strategically devised to elicit synergistic effects, ultimately leading to improved clinical outcomes [5]. Furthermore, an additional study [6] delineated a correlation between a favorable tumor response and improved survival. The exploration of innovative treatment regimens with the capacity to induce superior tumor responses presents a promising avenue for potentially improving survival outcomes in patients with esophagus cancer.

Recent studies on neoadjuvant chemoradiotherapy for esophageal cancer have underscored the importance of comparative assessments of different chemotherapeutic agents to optimize treatment protocols. These studies have explored diverse drug combinations and dosage regimens, aiming to identify the most effective therapeutic approach that enhances tumor response while minimizing the adverse effects [7,8,9,10,11,12,13,14]. The efficacy of various chemotherapy agents, such as cisplatin, 5-fluorouracil, paclitaxel, and docetaxel, has been under investigation regarding their potential roles in chemoradiotherapy [7,8,9]. Xi et al. [15] demonstrated that the combination of docetaxel with cisplatin yielded promising outcomes in patients who had esophageal cancer.

Among the diverse chemotherapeutic agents under investigation, docetaxel has emerged as a particularly promising contender in neoadjuvant chemoradiotherapy for esophageal cancer. A previous study reported the potential efficacy of docetaxel in neoadjuvant treatment strategies for esophageal cancer [16]. Chen et al. [14] observed that incorporation of docetaxel into concurrent chemoradiotherapy led to increased rates of pathologic complete response and improved survival outcomes among patients with locally advanced esophageal squamous cell carcinoma. Moreover, the safety profile of docetaxel in esophagus cancer has been demonstrated in several studies [17,18,19,20].

Previous studies [21,22] have demonstrated the clinical advantages of using docetaxel in patients diagnosed with gastro-esophageal cancer. The objective of this phase II clinical trial was to evaluate the effectiveness and safety of chemoradiotherapy employing the docetaxel, cisplatin, and 5-fluorouracil (DCF) regimen in patients with esophageal cancer.

## 2. Materials and Methods

### 2.1. Patients

The inclusion criteria were as follows: 1. histological confirmation of the squamous cell carcinoma of esophageal cancer or the adenocarcinoma of esophagogastric junction cancer (Siewert type I, and II), at stage T3N0 or T1-3N+ or T4Nx by AJCC, 2. age between 35 to 70 years old, 3. performance status of ECOG 0 or 1, 4. adequate hematopoietic function (neutrophil count is greater than or equal to 1500/mm^3^ and platelet count is greater than or equal to 100,000/mm^3^), 5. adequate hepatic function (AST is less than or equal to 1.5 times ULN, ALT is less than or equal to 1.5 times ULN, alkaline phosphatase is less than or equal to 2.5 times ULN, the ICG test is less than 15% or less than grade C of Child–Pughs classification, and bilirubin is less than or equal to 1.5 times ULN), 6. adequate renal function (GFR is greater than or equal to 60 mL/min), and 7. willingness to sign the informed consent form prior to undergoing the treatment procedure.

The exclusion criteria were as follows: 1. patients with stage T1-2N0 or inoperable T4 esophagus cancer, 2. patients with distant metastasis or carcinoma of the cervical esophagus, 3. a Forced Expiratory Volume in one second (FEV1) that is less than 1 L, 4. inadequate cardiovascular function (New York Heart Association class III or IV congestive heart failure, unstable angina pectoris, myocardial infarction within the past 3 months, significant arrhythmias, and other severe or uncontrolled cardiovascular disease), 5. pregnant or breast feeding women; fertile women of childbearing potential unless using a reliable and appropriate contraceptive method throughout the treatment period and for 12 months after the practice treatment, 6. concomitant illness that might be aggregated by chemotherapy or interfere with the practice assessment, such as hepatitis B and hepatitis C, HIV, infectious tuberculosis, or other active, non-controlled diseases such as congestive heart failure, ischemic heart disease, uncontrolled hypertension or arrhythmia, unstable diabetes mellitus, active peptic ulcers and autoimmune disease, 7. prior or concurrent malignancy except non-melanoma skin cancer or adequately treated head and neck cancer without metastasis or recurrence in two years and adequately treated carcinoma in situ of the cervix, 8. preexisting peripheral neuropathy greater than grade 1, 9. patients with significant neurologic or psychiatric disorders, including psychotic disorders, dementia, or seizures, 10. patients who have received prior chemotherapy, 11. patients who have received prior radiotherapy to the chest or to the head–neck region or to the abdomen, 12. patients who have received other concurrent experimental drugs or it has been less than 30 days since prior treatment for another clinical treatment, 13. patients with definite contraindications to corticosteroids as premedication, and 14. patients with a history of severe hypersensitivity reactions to any ingredient of the treatment drugs.

### 2.2. Treatment Protocol

The chemotherapy regimen consisted of docetaxel 35 mg/m^2^ combined with cisplatin 35 mg/m^2^ on day 1 and 5-FU 400 mg/m^2^ on day 1–4 in the first cycle, followed by cisplatin 35 mg/m^2^ on day 29, and 5-FU 400 mg/m^2^ on day 29–32.

The delineation of target volumes involved the definition of the gross tumor volume (GTV) as the tumor within the esophagus and any enlarged regional lymph nodes visible on the CT scan, endoscopic ultrasound (EUS) or FDG-PET. The clinical target volume (CTV) encompasses the GTV plus 3–5 cm margins superiorly and inferiorly, and 1.0 cm anteriorly and laterally. Lymph node regions with a probability of 10% or more of being microscopically invaded were included in the PTV. The supraclavicular nodes were included in the treatment fields for primary tumors located above the carina. The celiac trunk nodes were included for primary tumors located below the carina. The planned target volume (PTV) was generated from the CTV plus 0.5 cm margins in all directions to cover the setup error and internal organ motion. Patients received radiation at a dose of 180 cGy per fraction, with 5 fractions per week, and a total of 25 fractions for the PTV. Dose constraints for normal organs included 20 Gy to no more than 25% of the lung volume (V_20_ < 25%), 30 Gy to no more than 35% of the heart volume and 44 Gy to no more than 0% of the spinal cord volume. The RT treatment was planned by RapidArc treatment planning and delivered by Linear Accelerator.

Dose modifications were determined based on the severity of toxicity graded according to the common toxicity criteria of the cancer therapy evaluation program of Common Terminology Criteria for Adverse Events (CTCAE) version 4.0. For those toxicities considered by the investigator to be unlikely to become serious or life-threatening and that did not result in a delay or interruption of therapy (e.g., alopecia, altered taste etc.), treatment was continued at the same dose without a reduction or interruption. No dose reductions or interruptions were required for anemia as this can be satisfactorily controlled by transfusions. Any dose delay as per the investigator’s discretion was limited to a maximum of 2 weeks. 

### 2.3. Survey and Surgery

After the completion of neoadjuvant chemoradiotherapy, the patients underwent restaging examinations to determine their suitability for surgical intervention. These restaging examinations should be conducted as per the investigator’s discretion including physical examination, CBC, liver function tests, lung function tests (FEV1), biochemistry, CT of the chest and abdomen, PET scan, bone scan, and endoscopic evaluation with EUS. Results of the restaging examination that indicated no progression of disease allowed the patient to proceed with surgery.

After the completion of neoadjuvant chemoradiotherapy, tumor response assessment was conducted within 3 weeks for restaging purposes. Surgery was then performed within four to eight weeks after the last administration of radiotherapy.

### 2.4. Adjuvant Therapy

For patients with pathologic reports indicating Tany N2-3, an additional 2 cycles of the same chemotherapy regimen was given 6–8 weeks after surgery. These treatments followed clinical practice to treat eligible patients for the scientific evaluation of clinical experience.

### 2.5. Toxicity Assessment

Acute toxicity resulting from concurrent chemoradiotherapy was assessed weekly using the CTCAE v4.0. Physicians evaluated and recorded any adverse events weekly.

### 2.6. Statistical Analysis

The primary endpoints included the pathological complete response rate. The second endpoints were overall survival (OS) and disease-free survival (DFS). The OS was calculated from the date of diagnosis to the date of death from any cause, or the date of last follow-up. The DFS was calculated from the date of diagnosis to the date of recurrence, or the date of last follow-up.

The radiotherapy dose was 45 Gy in 25 fractions. The protocols of chemotherapy were docetaxel 35 mg/m^2^ for 1 h on day 1 and day 29, cisplatin 35 mg/m^2^ for 1 h on day 1 and day 29, and 5-FU 400 mg/m^2^ for 24 h on day 1–4 and day 29–32. The patients who were accepted following the re-staging exam in 4–8 weeks after the last administration of radiotherapy. The primary endpoints were disease-free survival (DFS), overall survival (OS), and toxicity. Survival times were estimated using the Kaplan-Meier method. The primary analysis for safety and side effects was carried out using descriptive statistics.

The statistical analyses were performed using SPSS software (version 22.0).

The study was approved by the Institutional Review Board of Taichung Veterans General Hospital (IRB number: CF14221A)

This research was funded by TTY Biopharm (VGH-TC CEP_1406).

## 3. Results

### Patient Characteristics

At the beginning of this study, a total of 29 newly diagnosed esophageal cancer patients were enrolled in this prospective study between April 2015 and November 2017. However, five of these patients did not undergo surgical intervention and were subsequently excluded from the study. Finally, data from 24 patients were subjected to analysis for this study.

Table 1 presents the patient characteristics. The majority of the patients were male (95.8%, 23/24), with a median age of 55.5 years, ranging from 44 to 66 years. All patients exhibited a baseline Eastern Cooperative Oncology Group (ECOG) performance status of less than 2. Histological confirmation revealed that all patients were diagnosed with squamous cell carcinoma. Regarding tumor location, 3 patients had tumors in the upper third of the esophagus, 17 patients had tumors in the middle third of the esophagus, and 4 patients had tumors in the lower third of the esophagus.

The distribution of disease stages among the patients was as follows: 79.1% (19/24) of patients had stage III disease, 12.5% (3/24) had stage II disease, and 8.4% (2/24) had stage IVA disease. Following surgery, 11 patients (45.8%) achieved a pathologic complete response.

Within the cohort, eight patients received adjuvant chemotherapy based on cisplatin. During the follow-up period, 12 patients experienced disease recurrence and/or distant metastasis. Among these patients, six patients had local and/or lymph node recurrence, four patients developed lung metastasis, and two patients exhibited bone metastasis. Among the 12 patients with relapsed disease, 6 patients received cisplatin and 5FU as salvage treatment, 2 patients underwent cisplatin, 5FU, and epirubicin as salvage treatment, 1 patient received Taxol as salvage treatment, 1 patient was treated by local radiotherapy as salvage treatment, 1 patient received cisplatin, 5FU, and immunotherapy as salvage treatment, and 1 patient was treated with a combination of cisplatin, 5FU, epirubicin, and immunotherapy as salvage treatment.

At the end of the follow-up period, two patients developed secondary cancers. One of these patients was diagnosed with *glioblastoma* multiforme, and the other was diagnosed with hypopharygeal cancer. Both of these patients died due to their secondary cancers.

Table 2 outlines the side effects observed in the study. The most commonly reported side effects included dysphagia, mucositis, anemia and leukopenia. The majority of patients experienced side effects at either grade 1 or grade 2 toxicity levels. Only one patient experienced grade 3 dysphagia. No occurrences of grade 4 or 5 toxicity were observed.

**Figure 1:**
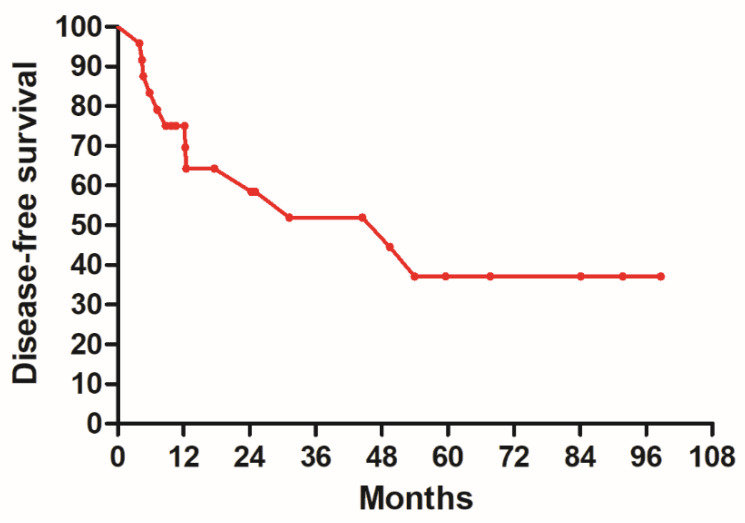
Disease-free survival of all patients.

**Figure 2:**
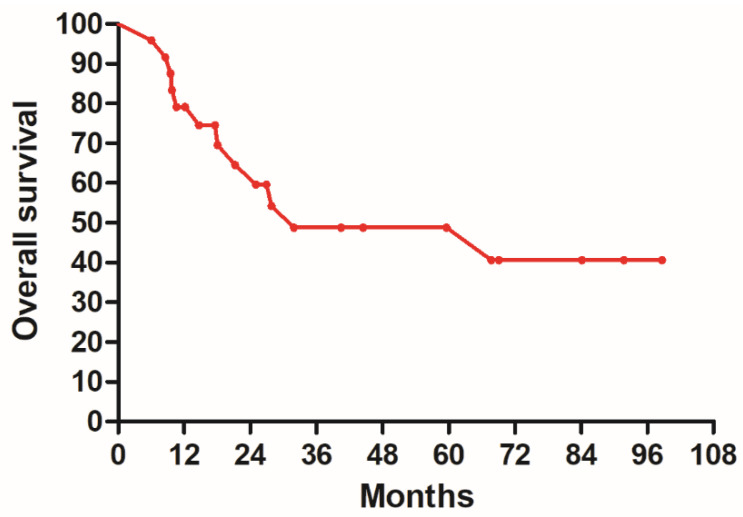
Overall survival of all patients.

The 5-year DFS was 37.1%, and the 5-year OS was 48.7%. Figure 1 shows the DFS and Figure 2 illustrates the OS.

## 4. Discussion

The neoadjuvant chemotherapy regimens under discussion for esophageal cancer include carboplatin plus Taxol. Over the past few decades, there has been numerous of proposals aimed at enhancing the treatment outcomes of esophageal cancer. These proposals encompass exploring diverse combined chemotherapy regimens and optimizing the timing of administration, whether it be before, after, or both before and after surgery. Docetaxel has been incorporated into chemotherapy regimens, and its efficacy and safety have been demonstrated in previous studies [17,18,19,20].

The CROSS trial [3,4] enrolled 178 esophageal cancer patients who received chemoradiotherapy utilizing a regimen of weekly carboplatin and paclitaxel in conjunction with radiotherapy (total dose of 41.4 Gy in 23 fractions), which was then followed by surgical intervention. The majority of patients (75%) of the CROSS trial were diagnosed with adenocarcinoma of the esophagus, with a predominant tumor location of the lower third (58%). Their finding revealed that leukopenia (60%) was the most common hematologic side effect. They also reported a pCR rate of 29%. The in-hospital mortality rate for the chemoradiotherapy–surgery group in the CROSS trial was reported as 4%. In our study, a monthly DCF regimen combined with radiotherapy (total dose of 45 Gy in 25 fractions) followed by surgery was employed. The most frequently observed hematologic toxicity of chemotherapy in our study was leukopenia, with 54% of patients experiencing grade 1 or grade 2 leukopenia. In this study, all patients were diagnosed with squamous cell carcinoma of the esophagus, with the majority of tumors located in the middle third (70%). We observed a pCR rate of 45.8% and no in-hospital mortality in our study. Comparing our findings to those of the CROSS trial, we noted similarities in the onset of leukopenia and achieved pCR rate. However, several differences were noted between the CROSS trial and our study. First, all patients in our study had squamous cell carcinoma of the esophagus and the majority of patients in the CROSS trial had adenocarcinoma of the esophagus. Second, the predominant tumor location of our patients was the middle third of the esophagus, whereas in the CROSS trial, most patients had tumors located in the lower third of the esophagus. These differences in the tumor pathology and location may contribute to variations in treatment response. Despite administering chemotherapy on day 1–4 and day 29–32, patients in our study experienced acceptable toxicity levels.

Pathologic complete response (pCR) serves as a widely recognized indicator for assessing the response to neoadjuvant chemoradiotherapy in esophagus cancer, and its correlation with prognosis is well established [23,24]. In our study, we conducted a comprehensive evaluation of treatment response using pCR, DFS and OS as key outcome measures. Only patients who underwent surgery and had available pathologic reports were included in the final analysis, ensuring robust assessment of treatment efficacy and outcomes.

Previous studies have investigated the efficacy of Taxotere in the treatment of esophagus cancer [20,25]. Nakamura et al. [20] demonstrated that the docetaxel, cisplatin, and 5FU (DCF) regimen yielded superior clinical outcomes compared to CF in neoadjuvant chemotherapy for patients with esophagus cancer. Matsuda et al. [25] used a propensity score-matched method to analyze the clinical outcomes of the DCF regimen in the real-world. Their findings provided evidence that neoadjuvant chemotherapy with the DCF regimen led to improved OS and recurrence-free survival compared to the CF regimen in patients with esophagus cancer. These studies highlight the potential of Taxotere in enhancing downstaging efforts for esophagus cancer. Sasaki et al. [26] analyzed 30 patients with esophageal squamous cell carcinoma who underwent neoadjuvant chemoradiotherapy with the DCF regimen, reporting a 3-year OS of 62.2%. In our clinical trial, we showed that the 5-year OS was 48.7%. These findings underscore the potential benefit of incorporating Taxotere-based regimens in the treatment of esophageal cancer, albeit with variations in reported survival outcomes across different studies.

Furthermore, Sasaki et al. [26] employed the DCF regimen in neoadjuvant chemoradiotherapy for advanced esophagus cancer and reported a pCR rate of 46% (14/30). In another study, Sasaki et al. [27] retrospectively reviewed 95 patients who underwent neoadjuvant chemoradiotherapy with either the CF regimen or the DCF regimen followed by surgery, and they revealed a higher pCR rate (38.6%) in the DCF arm compared to the CF arm. In our study, we achieved a comparable pCR rate of 45.8%, demonstrating consistency with precious findings regarding the effectiveness of the DCF regimen in inducing pCR in patients with esophageal cancer.

While the efficacy of the DCF regimen holds promise in the treatment of esophageal cancer, it is imperative to carefully consider the safety and tolerability of these treatment protocols. Treatment-related toxicities can significantly impact the patients’ quality of life and overall treatment outcomes. The DCF regimen is known to have a distinct toxicity profile, characterized by myelosuppression, neuropathy, and fluid retention. Studies by Sakai et al. [28] showed that grade 3 or 4 acute toxicity included leucopenia (85.7%), neutropenia (78.5%), and febrile neutropenia (21.4%). Ajani et al. [29] conducted a randomized trial involving 445 patients with advanced gastric or gastroesophageal cancer, comparing the DCF arm and the CF arm. They demonstrated that patients receiving the DCF regimen experienced higher rates of grade 3–4 neutropenia and febrile neutropenia compared to those receiving the CF regimen. These findings underscore the significance of vigilant monitoring and proactive management of treatment-related toxicities in patients undergoing neoadjuvant therapy with the DCF regimen. In our study, we demonstrated the tolerability of the DCF regimen. No grade 3 or 4 neutropenia was observed in our study. However, it is important to acknowledge that the severity of toxicity may be influenced by the doses of DCF administered. Therefore, identifying the optimal dose to achieve the best therapeutic outcomes with the fewest side effects is important.

The tolerance to treatment and the preservation of quality of life are considered in the selection of neoadjuvant treatment regimens. The decision between the DCF regimen and the CF regimen should be made after a thorough evaluation of individual patient factors, such as age, performance status, and any existing comorbidities. Tailoring the treatment approach to accommodate the specific needs and circumstances of each patient is essential for optimizing therapeutic outcomes while minimizing the impact of treatment-related side effects on their overall well-being. By prioritizing personalized care, we can strive to achieve the best possible balance between treatment efficacy and quality of life for patients undergoing neoadjuvant therapy for esophageal cancer. Future systemic treatments are poised to prioritize improved efficacy and reduced side effects. Immunotherapy emerges as one of the most promising treatments in this regard in the future. The ATTRACTION-3 trial [30] enrolled unresectable advanced or recurrent esophageal squamous cell carcinoma patients, comparing the treatment responses between immunotherapy and chemotherapy. In the ATTRACTION-3 trial, the choice of chemotherapy regimens was paclitaxel 100 mg/m^2^ once per week for 6 weeks, followed by 1 week off or docetaxel 75 mg/m^2^ every 3 weeks. This trial revealed an improvement in overall survival and improved safety with immunotherapy compared to chemotherapy in previously treated patients with advanced esophageal cancer. This may offer new hope for patients with recurrent or metastatic esophageal cancer. The CheckMate 648 trial [31] analyzed previously untreated, unresectable advanced, recurrent, or metastatic squamous cell carcinoma esophageal cancer patients. In the CheckMate 648 trial, they compared the combination of immunotherapy and chemotherapy with chemotherapy alone. Their finding revealed that the combination of immunotherapy and chemotherapy led to prolonged survival in unresectable advanced, recurrent or metastatic esophageal squamous cell carcinoma. These important studies showed the potential of immunotherapy to enhance treatment outcomes in esophageal cancer. However, it is important to note that the role of immunotherapy in neoadjuvant therapy in esophageal cancer is currently under study. In addition to considering treatment options such as immunotherapy, there are other significant factors that need to be addressed. One key consideration is the necessity to assess the expression of tumor cell programmed death ligand 1 (PD-L1) in tumor cells to determine the suitability of immunotherapy. This step ensures that patients receive personalized treatment tailored to their specific tumor biology, maximizing the possibility of therapeutic success. Furthermore, the cost associated with immunotherapy remains a notable challenge. The high expense of these new treatment regimens poses a barrier to accessibility for many patients, underscoring the need for continued efforts to reduce costs and improve affordability. Addressing these cost concerns is important to ensure equitable access to new treatment regimens for all patients with esophageal cancer. Overall, while immunotherapy provides a valuable addition to the treatment of esophageal cancer, addressing issues such as PD-L1 repression assessment and cost-effectiveness is essential to optimize its clinical use and maximize its impact on patient outcomes.

This study had several limitations. First, the relatively small sample size poses challenges in conducting subgroup analyses and identifying patients based on specific patient characteristics that may experience the most benefit from the DCF regimen treatment. Moreover, the predominance of male patients, constituting over 90% of patients in this study, raises questions about the regimen’s safety and efficacy across different genders. Second, the absence of a control arm also limits our ability to directly compare differences between various chemotherapy regimens. Third, the variability in salvage therapy received by patients can potentially confound the clinical outcomes. Fourth, the widespread use of carcinogens such as tobacco, alcohol, and betel nut among patients with esophageal cancer in our country may influence prognosis, including the risk of secondary cancers.

An advantage of this study was its rigorous methodology, wherein all patients underwent surgery, and their final pathologic reports were available to confirm the treatment response. This approach ensures a high level of certainty in the evaluation of treatment outcomes, enhancing the reliability of this study finding. However, to consolidate these results and establish the broader applicability of the DCF regimen in the management of esophageal cancer, further large prospectively clinical trials are needed to confirm the efficacy and safety of the DCF regimen in esophageal cancer patients who underwent neoadjuvant chemoradiotherapy followed by surgery.

## 5. Conclusions

The DCF regimen utilized in neoadjuvant chemoradiotherapy followed by surgery demonstrated tolerable toxicity profiles, with a notable 45.8% pCR rate and acceptable DFS and OS outcomes. The DCF regimen not only offers promising therapeutic benefits but also expands the chemotherapy regimens available to patients.

## Figures and Tables

**Table 1:** Patients’ characteristics (n = 24).

	Patient Number
Age	44–66
	Median: 55.5
Gender	
Male	23
Female	1
Site	
Upper third	3
Middle third	17
Lower third	4
Stage	
II	3
III	19
IVA	2
Response	
pCR	11
Non-pCR	13

Abbreviations: CR: complete response.

**Table 2:** Treatment-related toxicity.

Toxicity	Patient Number			
	Grade 1	Grade 2	Grade 3	Grade 4
Anemia	13	1	0	0
Leukopenia	6	5	0	0
Thrombocytopenia	3	0	0	0
Liver toxicity	3	1	0	0
Nausea/Vomiting	6	0	0	0
Diarrhea	4	0	0	0
Dysphagia	16	1	1	0
Mucositis	13	2	0	0
Dermatitis	7	2	0	0

## Data Availability

The data that support the findings of this study are available upon reasonable request from the corresponding authors. The data are not publicly available due to privacy or ethical restrictions.
